# On Accelerating Substrate Optimization Using Computational Gibbs Energy Barriers: A Numerical Consideration Utilizing a Computational Data Set

**DOI:** 10.1021/acsomega.3c09066

**Published:** 2024-01-29

**Authors:** Hiroaki Okada, Satoshi Maeda

**Affiliations:** † Graduate School of Chemical Sciences and Engineering, Hokkaido University, Sapporo, Hokkaido 060-8628, Japan; ‡ Department of Chemistry, Graduate School of Science, Hokkaido University, Sapporo, Hokkaido 060-0810, Japan; § Institute for Chemical Reaction Design and Discovery (WPI-ICReDD), Hokkaido University, Sapporo, Hokkaido 001-0021, Japan; ∥ ERATO Maeda Artificial Intelligence for Chemical Reaction Design and Discovery Project, Hokkaido University, Sapporo, Hokkaido 060-0810, Japan; ⊥ Research and Services Division of Materials Data and Integrated System (MaDIS), National Institute for Materials Science (NIMS), Tsukuba, Ibaraki 305-0044, Japan

## Abstract

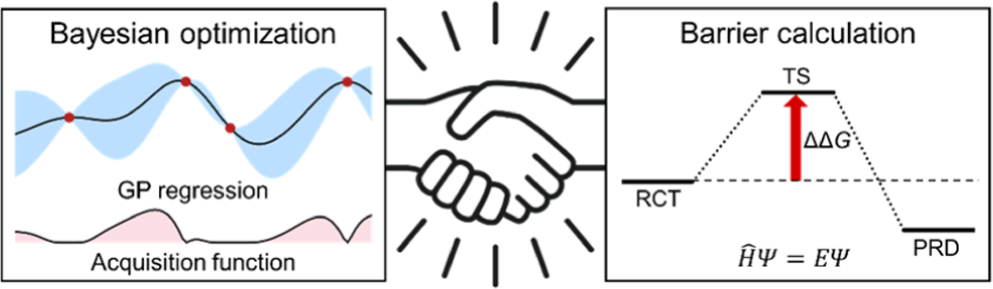

Substrate optimization is a time- and resource-consuming step in organic synthesis. Recent advances in chemo- and materials-informatics provide systematic and efficient procedures utilizing tools such as Bayesian optimization (BO). This study explores the possibility of reducing the required experiments further by utilizing computational Gibbs energy barriers. To thoroughly validate the impact of using computational Gibbs energy barriers in BO-assisted substrate optimization, this study employs a computational Gibbs energy barrier data set in the literature and performs an extensive numerical investigation virtually regarding the Gibbs energy barriers as virtual experimental results and those with systematic and random noises as virtual computational results. The present numerical investigation shows that even the computational reactivity affected by noises of as much as 20 kJ/mol helps reduce the number of required experiments.

## 1 Introduction

Substrate optimization is a time- and resource-consuming step in organic synthesis. Since the number of possible conditions increases exponentially depending on the number of substrates that need to be optimized, it is challenging to assess all of the combinations experimentally. It is generally done based on a chemist’s intuition and experience. Such an empirical process often takes a long time and consumes considerable resources. Thus, an efficient and reliable optimization method has been desired.

Informatics techniques such as linear and nonlinear regression, machine learning, data clustering, and black-box optimization, among others, are currently used in chemistry for a variety of purposes. These include predicting reaction yields and selectivities, elucidating reaction mechanisms, planning synthetic routes, developing descriptors, suggesting improved reaction conditions, and designing materials.^1−18^ Bayesian optimization (BO) is a powerful tool that balances exploration and exploitation.^19,20^ In BO, Gaussian process (GP) regression predicts an objective variable and estimates its variance. Then, the next candidate to be evaluated is proposed via an acquisition function. BO has been applied to various problems in chemistry,^21−38^ including reaction optimization in organic synthesis.^39−42^

On the other hand, Gibbs energy barriers, calculated using quantum chemical calculations, have been widely used to interpret and explain known reactivities and selectivities of organic reactions.^43−53^ In addition, their ability to predict results in advance of experiments has also been successful in many examples.^8,54−61^ In one-step reactions, the Gibbs energy difference between the transition state (TS) and the reactant determines the rate constant and thus represents the reactivity.^62^ Even in multistep reactions, the reactivity is represented by the Gibbs energy difference between the TS of the rate-determining step and the lowest intermediate before the rate-determining step, under the generalized pre-equilibrium approximation.^63,64^ Therefore, this study regards Gibbs energy barriers as experimental and computational reactivities.

**Figure 1:**
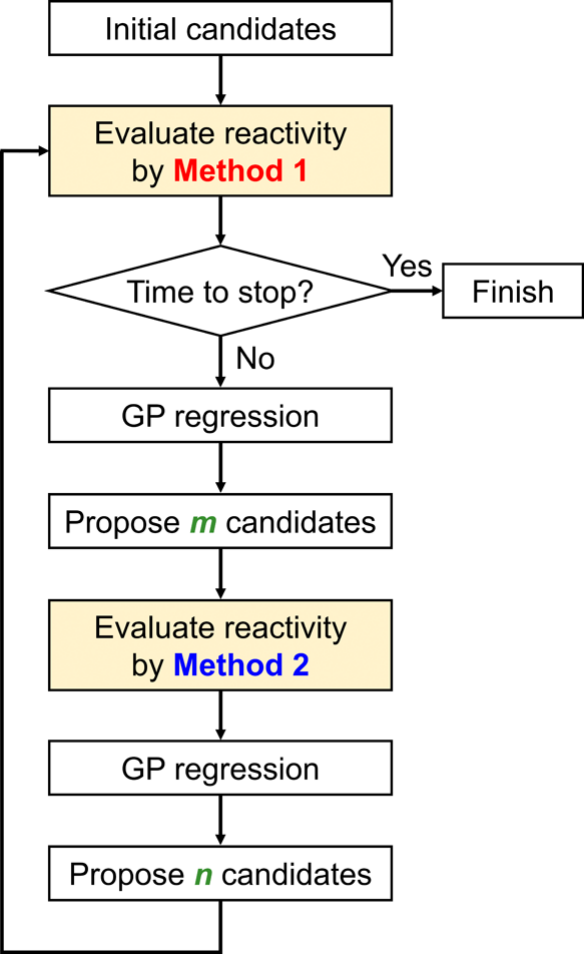
Flow of the BO-assisted substrate optimization using experimental and computational data. Method 1 and Method 2 correspond to the experiment and barrier calculation, respectively.

This study considers the use of theoretical Gibbs energy barriers to accelerate BO-assisted substrate optimization and, consequently, reduce the number of required experiments. In such a BO, data of two kinds, i.e., experimental data and computational data, are used, as illustrated in Figure 1. To do that, one must consider the deviation between computational and experimental results. Relying too much on the computational data misleads optimization due to computational errors. At the same time, suppressing the use of computational data too much does not help to reduce the number of experiments. Moreover, the deviation varies depending on the computational levels. In Gibbs energy barriers of organic reactions, the deviation typically is ∼5 kJ/mol by CCSD(T), 10–20 kJ/mol by density functional theory (DFT), and even more by semiempirical methods.

**Figure 2:**
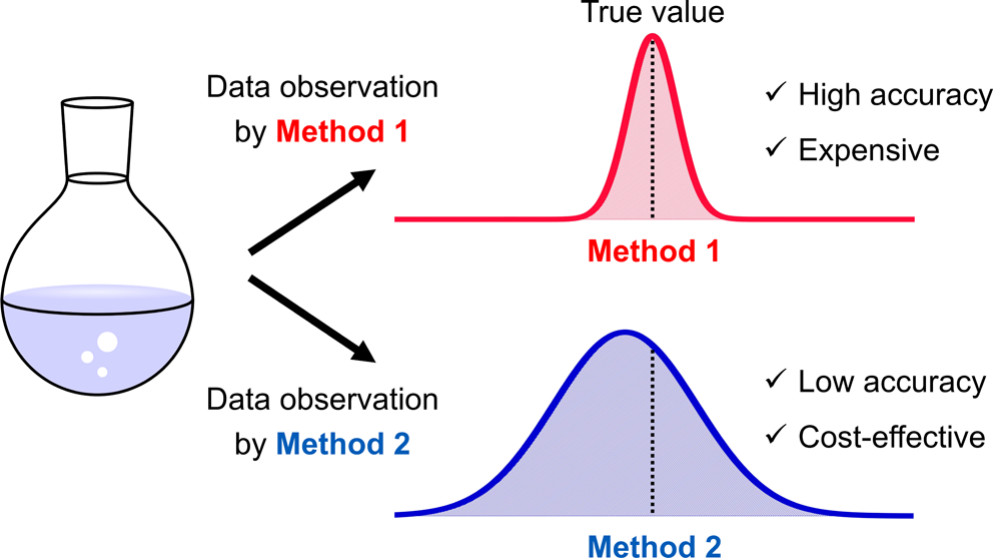
Two types of data observation approaches: Method 1 is a high accuracy and expensive observation; Method 2 is a low accuracy and cost-effective observation.

The main focus of this study, which examines the algorithm in Figure 1, is how to deal with the deviation between the two data sets of different nature (i.e., experimental vs computational). Figure 2 illustrates a similar situation where two measurement methods are combined to optimize a common value, one with high accuracy and high cost and one with low accuracy and low cost. The low-accuracy, low-cost measurement is also considered to be a biased and noisy but cost-effective source. Bayesian optimization that accesses biased and noisy but cost-effective sources of information along with accurate and expensive sources is called multi-information source Bayesian optimization.^65,66^ In the field of chemistry, multi-information source Bayesian optimization was employed to reduce the required cost of optimization and accelerate materials discovery,^67^ where it was demonstrated to be effective in the study of three optimization problems: global minimization of Rosenbrock function, geometry optimization of CO molecule, and maximization of intermolecular binding energies between hybrid organic–inorganic perovskites and solvent.

**Figure 3:**
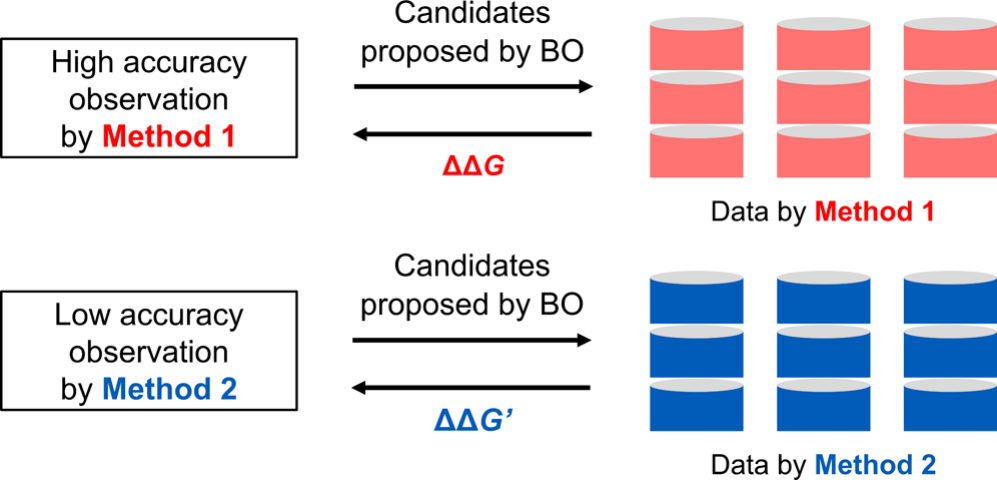
Virtual experiment (Method 1) and virtual computation (Method 2) in this study.

To investigate the performance of the algorithm in Figure 1 with sufficient statistical accuracy, avoiding enormous computational and experimental effort, we propose a procedure to do this virtually with practical effort instead of actually performing experiments and computations. Such a virtual investigation requires two sets of reactivity data. As mentioned above, the Gibbs energy barrier is considered as an index of reactivity throughout this paper. As discussed above, computational data based on quantum chemical calculations have errors due to the approximations used to solve the electronic wave equations, and thus can be considered a biased and noisy but cost-effective source of multi-information source Bayesian optimization. In this study, it is assumed that the experimental error bar is much smaller than the errors in quantum chemical calculations. With these assumptions, we construct a model, as shown in Figure 3. In this model, we first adopt a large set of Gibbs energy barrier data (*vide infra*). Another data set is created by replicating the original one and then modifying its values by adding random and systematic errors. The search for the optimal (low barrier) data in the original data set is then performed with reference to these two data sets. In such a virtual study, the original data set without errors is considered to be the virtual experimental data set, and the replicated and modified data set with errors is considered to be the virtual computational data set. It should be emphasized that the purpose of this study is not to find a good chemical reaction in a particular system. The purpose is to study the algorithm in Figure 1 using data of two different accuracies and to see how effective the algorithm is in reducing the number of references to the higher accuracy data.

**Figure 4:**
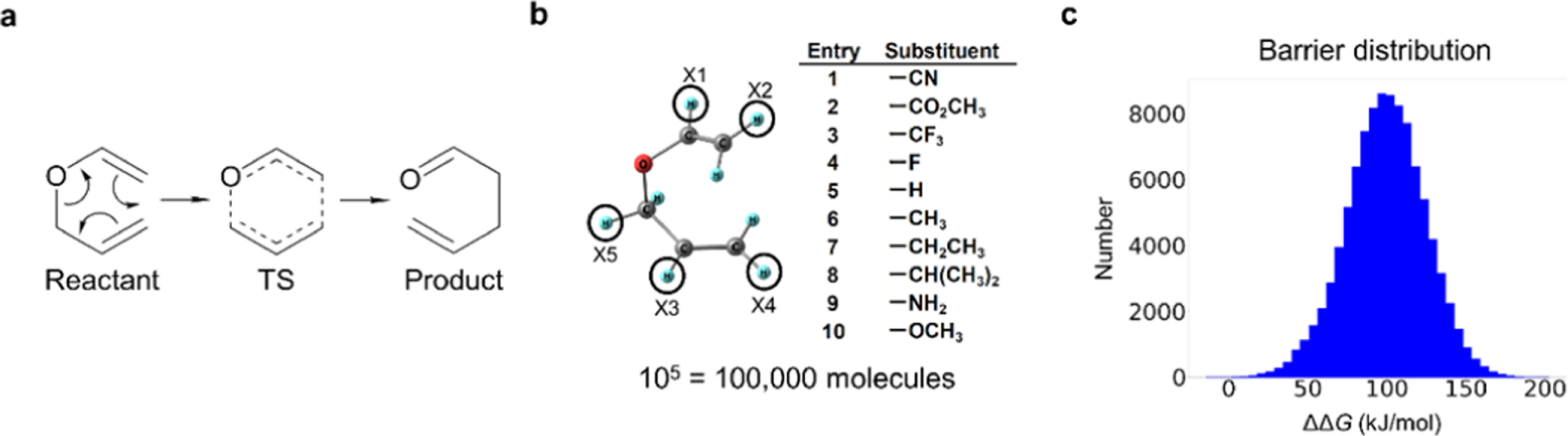
(a) Claisen rearrangement, (b) substitution sites and substituents considered in the Claisen rearrangement barrier data set,^68^ and (c) barrier distribution in the Claisen rearrangement barrier data set.

As a case study, this study focuses on the Claisen rearrangement and uses its theoretical Gibbs energy barrier data set based on semiempirical PM7 theory reported in the literature.^68^ The data set contains Gibbs energy barriers of the Claisen rearrangement of allyl vinyl ether (Figure 4a) with ∼100,000 different substitutions (Figure 4b). Even though all of the barriers are for the Claisen rearrangement, these barriers have a wide distribution, as illustrated in Figure 4c. It is emphasized again that the purpose of this study is not to predict a new reaction using the Gibbs barrier data set as descriptors, nor to provide an accurate data set for any particular system; the purpose is to examine the Bayesian optimization algorithm that combines two data sets with different accuracies. For this purpose, these data sets can be anything as long as they are not too chemically unrealistic. Although the data set we employed is based on semiempirical PM7 theory, it was carefully examined in the former publication (ref (68)) and was shown to well describe the substituent dependence of the reactivity of the Claisen rearrangement. Therefore, the following numerical experiments using the PM7 data set are sufficient for the present purpose.

This study conducts an extensive numerical investigation regarding these Gibbs energy barriers as virtual experimental data, while these Gibbs energy barriers plus systematic and random noises are virtual computational data, as illustrated in Figure 3. Such a numerical investigation avoids additional experimental and computational efforts, enabling massive trials to check statistical behaviors while adjusting various parameters. The present numerical investigation shows that even computational data including systematic and random errors accelerates BO-assisted substrate optimization and helps reduce the number of experiments.

## 2 Methods

### 2.1 Barrier Data Set Utilized

To perform virtual experiments and computations, we use the Gibbs energy barrier data set of the Claisen rearrangement.^68^ The Claisen rearrangement is a [3, 3] sigmatropic rearrangement of an allyl vinyl ether, as illustrated in Figure 4a. This data set includes theoretical Gibbs energy barriers of the Claisen rearrangement for 100,000 molecules computed based on a semiempirical theory PM7.^69^ Each molecule is generated by substituting 5 hydrogen atoms of the unsubstituted allyl vinyl ether molecule with 10 substituents. Five substitution sites and 10 substituents considered in the data set are summarized in Figure 4b. Although there are 928 missing values in the data set, these values were filled by random forest regression. It should be noted that the deviation between the PM7 data set and the more accurate computational data set obtained by, e.g., DFT is not essential in the present numerical investigation, which focuses purely on the deviations between the original data set and the replicated and modified data set. The only requirement for the data set used in this study is that it is chemically reasonable. The PM7 data set has been carefully studied in the literature and has been shown to reproduce well the chemical trends discussed in previous DFT studies.^70,71^ Furthermore, after extensive numerical analysis, it has been shown that the data set reflects well the chemical trends of different substitutions and is useful for testing different implementations of an optimization algorithm.^68^

In BO and random forest regression, each substituent is represented by a descriptor variable. As descriptor variables, eight parameters, HOMO energy, LUMO energy, HOMO–LUMO gap, NBO charge, Hammett constant,^72^ and Sterimol parameters (substituent length: L, minimum radius: B_1_, and maximum radius: B_5_), of each substituent capped by a hydrogen atom are used in this study. The reactivity of the Claisen rearrangement depends on the partial charge of the transition state, the stability of the product, and the interaction of the frontier orbitals. Previous studies have reported that these factors are controlled by substituent effects.^70,71^ Therefore, it is expected that the energies of the substituent frontier orbitals and the NBO charge will correlate with the reaction barrier. The Hammett constant, which represents the electron-donating or electron-withdrawing property of a substituent, and the Sterimol parameter, which represents the steric hindrance of a substituent, are also thought to affect reactivity. Since each aryl vinyl ether molecule in the data set has 5 substitution sites, each molecule is represented by 40 descriptor variables. Values of these parameters are listed in Table S1 in the Supporting Information.

### 2.2 Input and Output

BO is a sequential global optimization strategy to find x* that maximizes or minimizes output *f*(**x)**. In substrate optimization, input **x** is a set of descriptor variables representing a substrate combination. The output *f*(**x)** is an objective value such as the reaction yield, rate constant, and Gibbs energy barrier. In this study, **x** is a set of descriptor variables representing an allyl vinyl ether molecule and *f*(**x)** is the Gibbs energy barrier of their Claisen rearrangement.

In the present numerical investigation, a virtual experiment returns the Gibbs energy barrier for the corresponding allyl vinyl ether, i.e., ΔΔ*G*, from the data set. On the other hand, a virtual computation returns the Gibbs energy barrier for the corresponding allyl vinyl ether from the data set after adding systematic and random noises ϵ, i.e., ΔΔ*G* + ϵ, where ϵ is the Gaussian noise following the normal distribution  with mean μ and variance σ^2^. In quantum chemical calculations, a specific computational method often systematically underestimates or overestimates the Gibbs energy barriers for a particular chemical transformation. The former μ represents such a systematic deviation. The latter σ^2^ expresses deviations caused by factors having no systematic trend. It is noted that precisely representing a deviation for a specific computational level is not the purpose of this study. Instead, this numerical investigation aims to show that such virtual computational results can help reduce the number of virtual experiments.

### 2.3 Implementation of BO

Figure 1 presents our implementation of BO utilizing both virtual experimental (Method 1) and computational (Method 2) results. First, Gibbs energy barriers are evaluated by virtual experiments for *n* allyl vinyl ether molecules, where these are randomly chosen in the initial cycle or otherwise are ones proposed in the last cycle. The Gibbs energy barrier data are standardized to have a mean of 0 and a standard deviation of 1.

Second, a posterior mean and variance for the reaction barrier, representing its expected value and the uncertainty of the prediction, respectively, are estimated by GP regression. In the implementation of GP, a Python package GPyTorch is used.^73^ Given a set of observations
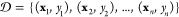
where
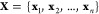
is the training input and
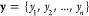
is the corresponding output, the mean μ function at point x* is given by
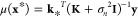
where k_*_^T^ is the vector of covariance function evaluations between x* and each training input
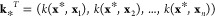
and **K** is the *n* × *n* covariance matrix of the training inputs **X** obtained using a kernel function *k*(**x**_*i*_,**x**_*j*_), σ_*n*_^2^ is the noise variance, and **I** is the identity matrix.

The variance σ^2^ function at point x* is given by
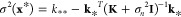
where k_**_ is the covariance function evaluation at x* itself
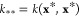
In this study, the radial basis function (RBF) kernel is employed as the kernel function given by
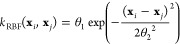
where θ_1_ is the output scale parameter and θ_2_ is the length scale parameter. These hyperparameters determine the shape of the covariance function and need to be optimized to fit the observations . This operation is performed via the type-II maximum a posteriori estimation. In this step, a γ priori is assigned over each hyperparameter. The parameter values of the γ prior for each hyperparameter are summarized in Table S3 in the Supporting Information. Then, *m* allyl vinyl ether molecules to be evaluated next by virtual computations are proposed via an acquisition function. As the acquisition function, we adopt the expected improvement (EI) function
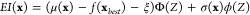
where μ(**x**) and *f*(**x**_*best*_) are a posterior mean predicted by GP regression and the function value of the current optimal solution, respectively, ξ is a parameter that controls the trade-off between exploitation and exploration and is set to 0.01 in this study,^19,39^ Φ(*Z*) and ϕ(*Z*) denote the cumulative density function and the probability density function of the standard normal distribution, respectively, and *Z* is expressed as
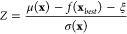
where σ(**x**) is a posterior standard deviation. The best candidate is estimated by evaluating EI values for all possible allyl vinyl ether molecules, where candidates that a virtual experiment or computation has already done are excluded to avoid duplication. In our initial tests, the EI function outperformed the other acquisition functions such as the probability improvement function and the upper confident bound function for the present system. Adopting the Kriging believer algorithm,^20^ the GP regression and the subsequent candidate proposal are iterated *m* times. In the first iteration, all previously evaluated virtual experimental and computational results are used with the same weight as data points for the GP regression. In the *k*th iteration, in addition to the virtual experimental and computational values, (*k* – 1) GP predicted mean values obtained in the first to (*k* – 1)-th iterations for the candidates proposed in the corresponding iterations are additionally used with the same weight as data points for the GP regression. In our initial tests, this Kriging believer algorithm outperformed the other multiple candidate suggestion schemes, such as the ε-greedy and Thompson sampling for the present system.^74,75^

Third, Gibbs energy barriers are evaluated by virtual computations for the proposed *m* allyl vinyl ether molecules. Then, *n* candidate molecules are proposed based on the GP regression through the same procedure taken in the second step, where candidates that the virtual experiment has already done are excluded to avoid duplication. The cycle is terminated after acquiring 100 virtual experimental results in this study.

## 3 Results and Discussion

To clarify whether computational data can be used to reduce the number of experiments, we benchmarked the performance of the algorithm in Figure 1. The success rate of finding one of the 10 most reactive allyl vinyl ether (one of those having the 10 lowest Gibbs energy barriers) within 100 virtual experiments was evaluated to benchmark the performance. The numerical investigations were done for the case without virtual computation (*m* = 0) and for all of the possible combinations of the virtual computational batch size *m* = 5, 10, 15, 20, 25, and 30, mean deviation μ = −20.0, –10.0, 0.0, 10.0, and 20.0 kJ/mol, and standard deviation σ = 0.0, 5.0, 10.0, 15.0, and 20.0 kJ/mol. In all cases, the virtual experimental batch size *n* was fixed to 5. We performed these numerical investigations 200 times for each combination, given different initial candidates and Gaussian noise ϵ, and discussed their averages as statistically (nearly) converged results. In total, 30,000 numerical investigations were thus performed. Using the literature data set enabled such a massive test requiring millions of experiments.

**Figure 5:**
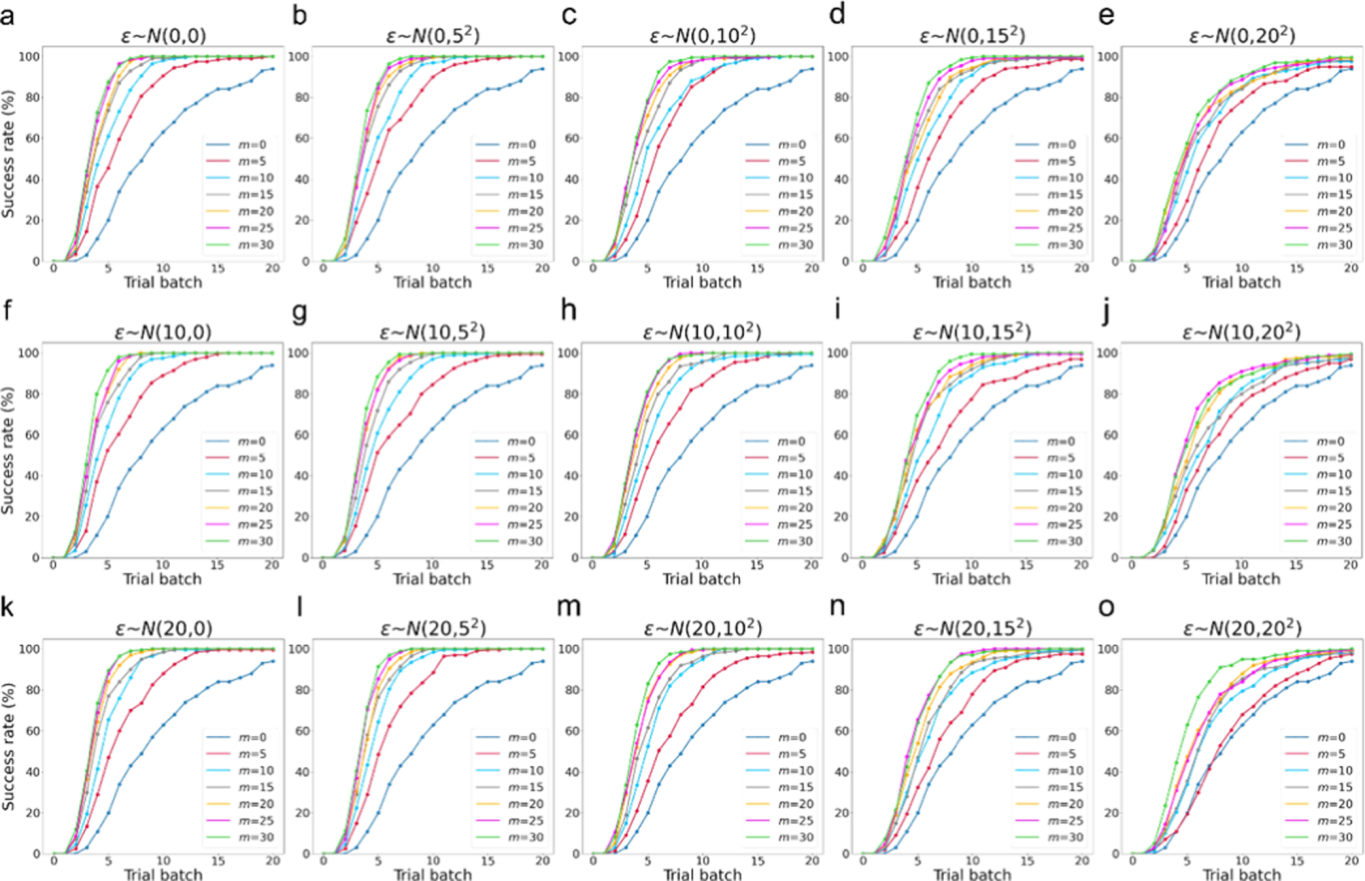
Rise in the success rate depending on the increase in the number of batches under each combination of *m*, μ, and σ.

Figure 5 shows the changes in performance for different combinations of *m*, μ, and σ. In each panel of Figure 5, the blue line denoted by *m* = 0 corresponds to the reference obtained without the use of virtual computational results. All of the other lines in Figure 5 show better performance than the reference. Even the worst μ = 20.0 and σ = 20.0 kJ/mol case in panel o shows a better success rate at the 20th batch (100th virtual experiment) than the reference. Knowing that even virtual computational results including such a significant error help improve the success rate and consequently reduce the number of virtual experiments is encouraging. It was also found that the success rate decreases compared to the reference when μ and σ exceed the range |μ| ≤ 20 kJ/mol and σ ≤ 20 kJ/mol, as shown in the Supporting Information (VI). That is, whether the present method works well depends on the size of the errors.

Figure 5 also shows that the performance changes, depending on *m* and σ. When *m* increases, the success rate rises rapidly. This is an intuitively reasonable result that using more virtual computational data improves performance. When σ increases from the left panel to the right panel in Figure 5, the success rate decreases. In contrast, μ does not affect the performance much compared to *m* and σ. This implies that the GP regression easily recognizes and learns the systematic deviation. Similar trends are also found for μ = −10 and −20 kJ/mol cases, as shown in the Supporting Information (Figure S1).

**Figure 6:**
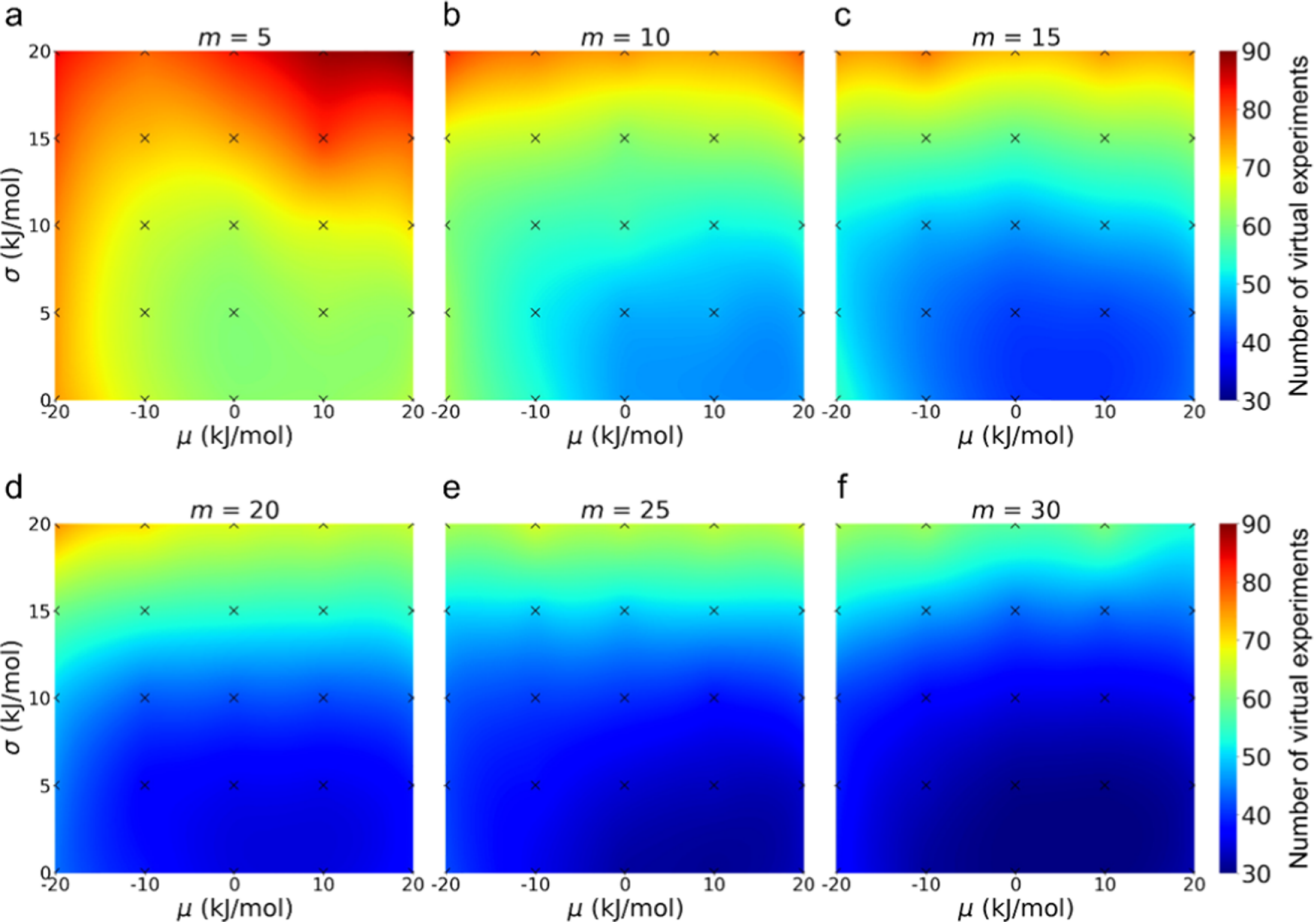
Heatmap of the number of virtual experiments required to reach the 95% success rate of finding the top 10 reactions from the Claisen barrier data set.

These trends are further visualized on the heatmaps in Figure 6 for different virtual computational batch sizes. Each heatmap presents the number of virtual experiments required to reach the 95% success rate. As *m* increases, the blue area in which the 95% success rate can be achieved by virtual experiments less than 50 times increases. Such a trend indicates that the number of experiments can be reduced using more computational results. As seen in Figure 5a, the improvement saturates when *m* is increased over a certain level, even with μ = σ = 0.0 kJ/mol. This would be due to the Kriging believer algorithm, which suggests multiple candidates using a GP model and emphasizes errors in the GP model.

Figure 6 also shows that the change in performance is highly dependent on σ but not so much on μ. However, all of the heatmaps are asymmetric and show better performance when μ is positive. This is because the virtual computational results bias the GP model to accept molecules like those already evaluated by virtual computation when μ is negative (underestimating their barriers), especially in the early optimization stage. On the other hand, the virtual computational results bias the GP model to avoid molecules like ones already evaluated by virtual computation when μ is positive (overestimating their barriers). Such a difference in the early stage explains the asymmetric heatmaps in Figure 6. Although numerical investigations were performed only using BO in this study, we expect similar results to be obtained even with other optimization algorithms such as the genetic algorithm and particle swarm optimization algorithm.

Finally, we discuss issues one needs to consider when one uses the algorithm in Figure 1. One is how to determine the rate-determining step from a multistep reaction profile and compute an overall reactivity.^76^ Another is how to accurately determine the ensemble of various conformations at the rate-determining TS.^45−52,77^ There also are methods to directly determine the reaction yield through automatic reaction path network explorations considering formations of various resting and byproduct states.^78,79^ Evaluating the reactivity accurately, considering an entire reaction profile or reaction path network, could often be more time-consuming than a single experiment. On the other hand, focusing only on a few elementary steps could mislead BO due to a wrong kinetic description based on an oversimplified model. In a similar vein, the choice of the computational level is also crucial. In general, cost and efficiency are a trade-off in quantum chemical calculations. However, using computational methods with lower computational costs can result in larger computational errors and weaken the superiority of our method. Therefore, when using our method, it is important to carefully determine the appropriate computational level for barrier calculations, taking into consideration the available computational resources and costs and taking note of a balance between the time and cost required for a single experiment and a single barrier calculation. While our method may reduce the number of experiments, it may not be as effective for reactions that require a significant computational effort to compute the reaction barrier. On the other hand, reactions explainable with a relatively simple mechanism possessing a clear rate-determining step would be suitable targets for the algorithm shown in Figure 1.

## 4 Conclusions

In this study, we virtually validated the impact of using computational Gibbs energy barriers in BO-assisted substrate optimization and showed that it potentially accelerates BO-assisted substrate optimization and reduces the number of required experiments. To perform a thorough numerical investigation, Gibbs energy barriers in the literature data set were employed virtually regarding the Gibbs energy barriers as experimental results and those with systematic and random noises as computational ones. Such a numerical model allowed us to examine as many as 30,000 cases and acquire statistically (nearly) converged outputs. The present numerical validation suggested that the use of computational Gibbs energy barriers considerably accelerates substrate optimization, especially when a computational method affected by a small random error is adopted. It was also found that systematic errors are not a serious matter. Moreover, it was shown that even a computational method affected by a relatively sizable random error of 20 kJ/mol, more significant than the typical error by DFT, is sufficiently helpful in reducing the number of experiments. These findings encourage the utilization of computational Gibbs energy barriers in actual substrate optimizations. Although this study has focused on the optimization of the combination of substituents in the reactants of the Claisen rearrangement, our method can easily be applied to reaction optimizations of different types, varying temperature, solvent, ligand on organometallic complex, etc. In these cases, barrier calculations are also carried out by changing these conditions. A further study combining real experimental and computational results is in progress in our group.

## Data Availability

All of the barrier data used in this study were taken from the Supporting Information of ref (68). The Python code used for implementing Bayesian optimization and benchmarking its performance can be found at: https://github.com/Hiroaki-Okada/bo-comp-barrier.

## Supporting Information Available

Substituent descriptors, substituent geometries, target solutions, hyperparameters of Bayesian optimization, results of other numerical experiments, and references (PDF)
