# Supplementary material for: On Accelerating Substrate Optimization Using Computational Gibbs Energy Barriers: A Numerical Consideration Utilizing a Computational Data Set
Source: ACS Omega. 2024 Jan 29;9(6):7123–31. doi: 10.1021/acsomega.3c09066 (PMC10870292; doi:10.1021/acsomega.3c09066)
Supplement: Supplementary file 1 — ao3c09066_si_001.pdf [file ao3c09066_si_001.pdf]

Supporting Information for:

On accelerating substrate optimization using computational Gibbs energy barriers: A numerical consideration utilizing a computational dataset

Hiroaki Okada[1], Satoshi Maeda[2-5]

[1]Graduate School of Chemical Sciences and Engineering, Hokkaido University; Sapporo Hokkaido 060-8628, Japan

[2]Department of Chemistry, Graduate School of Science, Hokkaido University; Sapporo, Hokkaido 060-0810, Japan

[3]Institute for Chemical Reaction Design and Discovery (WPI-ICReDD), Hokkaido University; Sapporo, Hokkaido 001-0021, Japan

[4]ERATO Maeda Artificial Intelligence for Chemical Reaction Design and Discovery Project, Hokkaido University; Sapporo, Hokkaido 060-0810, Japan

[5]Research and Services Division of Materials Data and Integrated System (MaDIS), National Institute for Materials Science (NIMS); Tsukuba, Ibaraki 305-0044, Japan

Table of contents

I. List of substituent descriptors ... 3

II. Geometries of substituents capped by a hydrogen atom at the *ω*B97X-D/def2-SVP level of theory ... 4

III. Chemical analysis of computational barrier ... 7

IV. Prior distribution of hyperparameters ... 8

V. Performances when the noise mean μ is negative ... 9

VI. Performances when the noise mean μ and standard deviation σ are large ... 10

VII. References ... 12

I. List of substituent descriptors

In this study, 8 descriptor variables were used to represent the electronic and steric differences among the substituents (Table S1). HOMO energy, LUMO energy, HOMO-LUMO gap, and NBO charge were calculated using the Gaussian 16 software package[1] by density functional theory at the ωB97X-D/def2-SVP[2,3] level of theory. Hammett constants σ_p were taken from the literature.[4] Sterimol parameters L, B_1, and B_5 were calculated using Winmoster.[5]

Table S1. Lists of descriptor variables for each substituent.

	HOMO (eV)	LUMO (eV)	Gap (eV)	NBO Charge	σ_p	L (Å)	B_1 (Å)	B_5 (Å)
CN	-12.27	2.58	14.85	0.22152	0.66	3.7810	1.7000	1.7000
CO_2CH_3	-10.13	2.25	12.38	0.13372	0.45	4.6592	1.7901	3.7086
CF_3	-13.11	4.38	17.49	0.12820	0.54	3.0327	2.0928	2.7158
F	-12.99	3.80	16.79	0.53020	0.06	2.3889	1.4700	1.4700
H	-13.86	4.25	18.11	0.00000	0.00	1.9578	1.2000	1.2000
CH_3	-12.86	4.27	17.13	0.21486	-0.17	2.7979	1.7171	2.2351
CH_2CH_3	-11.45	4.01	15.45	0.21158	-0.15	3.9584	1.7620	3.3698
$CH(CH_3)_2$	-11.00	3.81	14.81	0.21332	-0.15	3.9102	2.1241	3.3814
NH_2	-9.20	3.85	13.04	0.35184	-0.66	2.5695	1.5500	2.1817
OCH_3	-9.56	3.84	13.40	0.45253	-0.27	3.7011	1.5200	3.2946

II. Geometries of substituents capped by a hydrogen atom at the ωB97X-D/def2-SVP level of theory

H-CN

C	0.055147059000	-0.000000000000	0.060249009042
N	0.055147059000	0.000000000000	1.212596284765
H	0.055147059000	0.000000000000	-1.018338323806

H-CO_2CH_3

C	-0.396589378000	0.842657409756	-1.246304213004
O	-0.396589378000	1.845165955127	-0.592379219928
O	-0.396589378000	-0.395304097283	-0.760325205108
C	-0.396589378000	-0.514726820442	0.655755772208
H	0.496024896387	-0.039560468335	1.087766235896
H	-1.289203652387	-0.039560468335	1.087766235896
H	-0.396589378000	-1.588101724124	0.873841916708
H	-0.396589378000	0.802384319638	-2.354303861667

H-CF_3

C	-0.481422497256	-3.502325523165	-0.400871561615
F	-0.863664175153	-2.316664037189	0.059957507824
F	-1.317137106412	-4.426176658010	0.059954208181
F	0.736510390231	-3.764122748049	0.059928810023
H	-0.481402666410	-3.502330068586	-1.502744674413

H-F

F	0.000000000000	0.000000000000	-0.000513212995
H	0.000000000000	0.000000000000	-0.919486787005

H-H

H	-0.057571214000	0.000000000000	0.115062151458

H	-0.057571214000	0.000000000000	-0.642768297458

H-CH_3

C	0.224341519297	2.948721939804	-3.970496222662
H	-0.810719010893	2.939690992216	-3.604549523194
H	0.749695202114	2.056850944931	-3.604547287685
H	0.734047543817	3.849624871577	-3.604545957045
H	0.224338319666	2.948720332472	-5.068381174414

H-CH_2CH_3

C	0.069248443004	2.958790058522	-4.253379086962
H	0.956456486179	3.529910611924	-3.939781677911
H	-0.817959600199	3.529910611901	-3.939781677950
C	0.069248443000	1.556968403845	-3.656803590682
H	0.956448719198	0.985856893575	-3.970451706157
H	-0.817951833232	0.985856893606	-3.970451706120
H	0.069248443023	1.587579805205	-2.556473646670
H	0.069248443026	2.928044327422	-5.353733704548

H-$CH(CH_3)_2$

C	0.118887155926	3.182399975504	-4.952415155638
H	0.118887155914	4.259417661971	-4.714015659636
C	1.391851318330	2.542577023866	-4.407405575838
C	-1.154077006526	2.542577023854	-4.407405575966
H	-2.056368379590	3.022036734628	-4.815954156185
H	-1.200681728590	1.471347130805	-4.661360319643
H	-1.200790069019	2.623050879527	-3.309441594822
H	1.438456040423	1.471347130815	-4.661360319486
H	1.438564380730	2.623050879560	-3.309441594693
H	2.294142691424	3.022036734639	-4.815954155988
H	0.118887155978	3.118351344831	-6.053627644106

H-NH_2

N	-0.897788482014	2.118810216994	2.055012267734
H	-0.288419668773	2.931150993621	2.144815621709

H	-0.288419668752	1.306469440382	2.144815621695
H	-1.215335568457	2.118810217002	1.086277757865

H-OCH$_3$

O	-0.243795390878	3.554577913648	-5.019643929039
C	0.035375413696	2.484461595067	-4.162259738076
H	-0.737705669977	1.719060362238	-4.320790663534
H	1.015726707764	2.010879180317	-4.363134058085
H	0.007131502554	2.766300626576	-3.091986198147
H	0.426389336846	4.229858915160	-4.889186942116

III. Chemical analysis of computational barrier

The top 10 substitution combinations that minimized the reaction barrier in the dataset are shown in Table S2. Electron-donating groups are predominantly located at positions X1, X2, and X5, while electron-withdrawing groups are mainly present at positions X2 and X3. The reason why these substituents reduce the barrier can be explained from chemical and numerical perspectives such as partial charges of transition state and stabilization of products.[6–8] As described in the results section, we examined the number of virtual experiments required for our method to discover any of the top 10 combinations. The ability of Bayesian optimization to quickly discover these combinations means that the Gaussian process regression can recognize the chemical and numerical properties of the substituents that lower the barrier, and that the search is balanced between exploration and exploitation.

Table S2. Substituents at different substitution sites in the top 10 reactions in terms of $\Delta\Delta G$ in the barrier dataset. Electron-donating groups are written in red, electron-withdrawing groups in blue, and hydrogen and alkyl groups in black.

Ranking	X1	X2	X3	X4	X5
1	OCH_3	CN	CO_2CH_3	NH_2	NH_2
2	OCH_3	CF_3	CO_2CH_3	OCH_3	NH_2
3	OCH_3	CF_3	CO_2CH_3	NH_2	NH_2
4	NH_2	CN	CO_2CH_3	NH_2	NH_2
5	F	CF_3	CO_2CH_3	NH_2	NH_2
6	OCH_3	CO_2CH_3	CF_3	OCH_3	NH_2
7	F	CN	CO_2CH_3	NH_2	NH_2
8	OCH_3	CO_2CH_3	CH_3	OCH_3	NH_2
9	NH_2	CF_3	CO_2CH_3	NH_2	NH_2
10	OCH_3	CN	H	NH_2	NH_2

IV. Prior distribution of hyperparameters

In GPyTorch, one can assign a prior distribution over each hyperparameter. In this study, the gamma distributions were specified as priors. The gamma distribution is a type of continuous probability distribution and has two parameters called a shape parameter α and a rate parameter β. In our implementation, α and β for length scale parameter and output scale parameter of radial basis function kernel, and noise were specified as shown in Table S3.

Table S3. Lists of shape parameter α and rate parameter β for each hyperparameter.

Parameter	α	β
Length scale parameter	1.5	0.1
Output scale parameter	7.0	1.0
Noise	1.5	0.5

V. Performances when the noise mean μ is negative

Figure S1 shows the changes in performance for different combinations of m, μ, and σ when μ is negative. In each panel of Figure S1, the blue line denoted by m = 0 corresponds to the reference obtained without the use of virtual computational results. All of the other lines in Figure S1 show better performance compared to the reference.

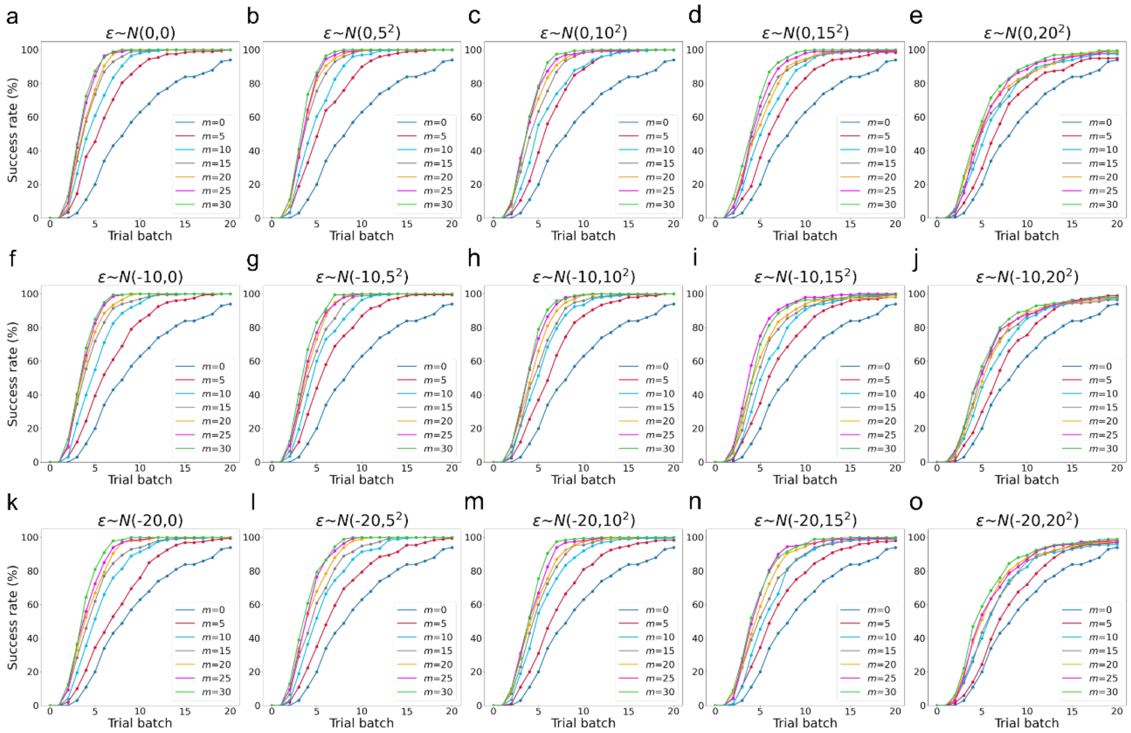

Figure S1. Rise in the success rate depending on the increase in the number of batches under different combinations of m, μ, and σ, when μ is negative.

VI. Performances when the noise mean μ and standard deviation σ are large

Figure S2 shows the changes in performance for different combinations of m, μ, and σ, when μ and σ are large. Specifically, the numerical investigations were done for the case without the use of virtual computation (m = 0) and for all of the possible combinations of the virtual computational batch size m = 5, 10, 15, 20, 25, and 30, mean deviation μ = 20.0, 30.0, and 40.0 kJ/mol, and standard deviation σ = 20.0, 25.0, 30.0, 35.0, and 40.0 kJ/mol. In each panel of Figure S2, the blue line denoted by m = 0 corresponds to the reference obtained without the use of virtual computational results. As shown in Figure S2, when the computational error is too large, our method cannot accelerate the convergence of Bayesian optimization and requires more virtual experiments than general Bayesian optimization. Therefore, when the computational error is too large, e.g. exceeding the range $|\mu| \leq 20$ kJ/mol and $\sigma \leq 20$ kJ/mol in the present case, our method does not work well.

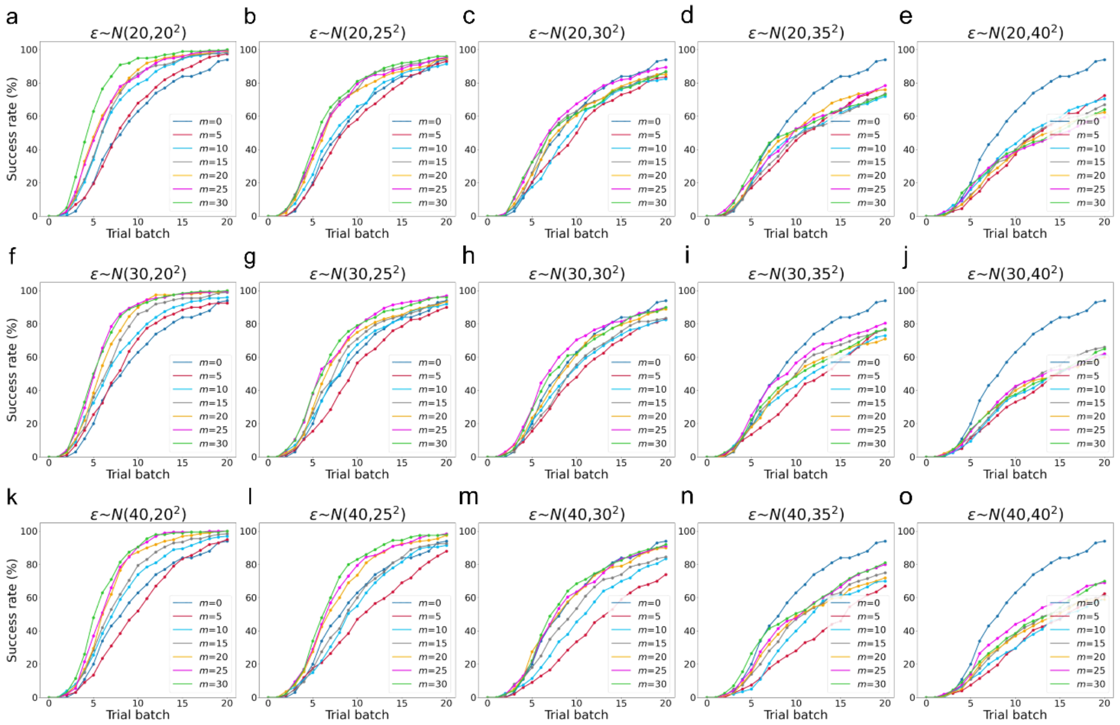

Figure S2. Rise in the success rate depending on the increase in the number of batches under different combinations of m, μ, and σ, when μ and σ are large.

VII. References

(1) Gaussian 16, Revision C.01, Frisch, M. J.; Trucks, G. W.; Schlegel, H. B.; Scuseria, G. E.; Robb, M. A.; Cheeseman, J. R.; Scalmani, G.; Barone, V.; Petersson, G. A.; Nakatsuji, H.; Li, X.; Caricato, M.; Marenich, A. V.; Bloino, J.; Janesko, B. G.; Gomperts, R.; Mennucci, B.; Hratchian, H. P.; Ortiz, J. V.; Izmaylov, A. F.; Sonnenberg, J. L.; Williams-Young, D.; Ding, F.; Lipparini, F.; Egidi, F.; Goings, J.; Peng, B.; Petrone, A.; Henderson, T.; Ranasinghe, D.; Zakrzewski, V. G.; Gao, J.; Rega, N.; Zheng, G.; Liang, W.; Hada, M.; Ehara, M.; Toyota, K.; Fukuda, R.; Hasegawa, J.; Ishida, M.; Nakajima, T.; Honda, Y.; Kitao, O.; Nakai, H.; Vreven, T.; Throssell, K.; Montgomery, J. A., Jr.; Peralta, J. E.; Ogliaro, F.; Bearpark, M. J.; Heyd, J. J.; Brothers, E. N.; Kudin, K. N.; Staroverov, V. N.; Keith, T. A.; Kobayashi, R.; Normand, J.; Raghavachari, K.; Rendell, A. P.; Burant, J. C.; Iyengar, S. S.; Tomasi, J.; Cossi, M.; Millam, J. M.; Klene, M.; Adamo, C.; Cammi, R.; Ochterski, J. W.; Martin, R. L.; Morokuma, K.; Farkas, O.; Foresman, J. B.; Fox, D. J. Gaussian, Inc., Wallingford CT, 2016.

(2) Chai, J.-D.; Head-Gordon, M. Long-Range Corrected Hybrid Density Functionals with Damped Atom–Atom Dispersion Corrections. *Phys. Chem. Chem. Phys.* **2008**, *10*, 6615–6620.

(3) Weigend, F.; Ahlrichs, R. Balanced Basis Sets of Split Valence, Triple Zeta Valence and Quadruple Zeta Valence Quality for H to Rn: Design and Assessment of Accuracy. *Phys. Chem. Chem. Phys.* **2005**, *7*, 3297–3305.

(4) Hansch, Corwin.; Leo, A.; Taft, R. W. A Survey of Hammett Substituent Constants and Resonance and Field Parameters. *Chem. Rev.* **1991**, *91*, 165–195.

(5) Winmostar V9, X-Ability Co. Ltd., Tokyo, Japan, 2019.

(6) Okada, H.; Maeda, S. A Dataset of Computational Reaction Barriers for the Claisen Rearrangement: Chemical and Numerical Analysis. *Mol. Inform.* **2022**, *41*, 2100216.

(7) Aviyente, V.; Yoo, H. Y.; Houk, K. N. Analysis of Substituent Effects on the Claisen Rearrangement with Ab Initio and Density Functional Theory. *J. Org. Chem.* **1997**, *62*, 6121–6128.

(8) Aviyente, V.; Houk, K. N. Cyano, Amino, and Trifluoromethyl Substituent Effects on the Claisen Rearrangement. *J. Phys. Chem. A* **2001**, *105*, 383–391.
